# Prognostic value of pan-immune-inflammation value in colorectal cancer patients: A systematic review and meta-analysis

**DOI:** 10.3389/fonc.2022.1036890

**Published:** 2022-12-22

**Authors:** Xiao-Chuan Yang, Hui Liu, Ding-Cheng Liu, Chao Tong, Xian-Wen Liang, Ri-Hui Chen

**Affiliations:** ^1^ Department of Interventional Radiology, Central South University, Xiangya School of Medicine Affiliated Haikou Hospital, Haikou, Hainan, China; ^2^ Department of Hepatobiliary Surgery, Hainan General Hospital, Haikou, Hainan, China

**Keywords:** colorectal cancer, pan-immune-inflammation value, overall survival (OS), progression-free survival (PFS), meta-analysis

## Abstract

**Background:**

The pan-immune-inflammation value (PIV) has been reported as a novel prognostic biomarker in multiple malignancies. The aim of this study is to investigate the prognostic value of the PIV in patients with colorectal cancer.

**Methods:**

We comprehensively searched electronic databases including PubMed, Embase and Web of Science up to August 2022. The endpoints were survival outcomes. Hazard ratios (HRs) with 95% confidence intervals (CIs) for survival data were collected for analysis.

**Results:**

Six studies including 1879 participants were included. A significant heterogeneity in the PIV cut-off value among studies was observed. The combined results indicated that patients in the high baseline PIV group had a worse overall survival (HR=2.09; 95%CI: 1.67-2.61; P<0.0001; I^2 =^ 7%) and progression-free survival (HR=1.82; 95%CI: 1.49-2.22; P<0.0001; I^2 =^ 15%). In addition, early PIV increase after treatment initiation was significantly associated with decreased overall survival (HR=1.79; 95%CI: 1.13-2.93; P=0.01; I^2 =^ 26%), and a trend toward poor progression-free survival (HR=2.00; 95%CI: 0.90-4.41; P=0.09; I^2 =^ 70%).

**Conclusion:**

Based on existing evidence, the PIV could act as a valuable prognostic index in patients with colorectal cancer. However, the heterogeneity in the PIV cut-off value among studies should be considered when interpreting these findings.

## 1 Background

Colorectal cancer is one of the most common malignancies in the world, accounting for about 10% of newly diagnosed cancers and cancer-related deaths (1). Despite significant advances in surgery-based multimodal therapy for colorectal malignancy, the prognosis of most patients, especially those with advanced stages, is still unsatisfactory (2–4). ​Consequently, it is essential to develop a useful prognostic index to predict postoperative recurrence and survival in colorectal cancers, aiming to formulate treatment plans for patients in the clinic.

Cancer-related inflammation is prevalent in most patients with malignancy, which can promote tumor progression and suppress treatment response (5, 6). Increasing evidence has reported that cancer-related inflammation plays an important role in postoperative recovery and prognosis of cancer patients (7, 8). Therefore, inflammation-based biomarkers are expected to be valuable predictors of surgical and long-term outcomes. For example, as the most common indicators of systemic inflammation, neutrophil (9), platelet (10) and monocyte (11) have been reported as strong indicators for increased postoperative complications, prolongation of hospital stays and poor survival outcomes in several types of malignancies. On the contrary, tumor-infiltrating lymphocyte subsets, such as CD8+ T cells and memory T cells, are associated with better prognosis in various tumors (12, 13).

In recent years, a novel biomarker, the pan-immune-inflammation value (PIV), which integrates peripheral neutrophil, platelet, monocyte and lymphocyte (neutrophil x platelet x monocyte/lymphocyte), has been reported as a promising predictor of long-term outcomes in cancers, because it can precisely reflect the inflammatory and immune status of patients with malignancy (14–17). A recent meta-analysis demonstrated that high PIV before treatment indicates poor prognosis in cancer patients (18). Nevertheless, the role of the PIV in survival outcomes of colorectal cancer remains inconclusive and no meta-analysis is available so far. In addition, emerging studies on the PIV and survival outcomes in colorectal cancer have been reported in recent years. Thus, we performed a systematic review and meta-analysis based on existing evidence to investigate the value of the PIV in long-term survival outcomes in patients with colorectal cancer.

## 2 Methods

### 2.1 Search strategy

The current study was performed in line with the Preferred Reporting Items for Systematic Reviews and Meta-Analyses (PRISMA) guidelines to identify studies that assess the association of PIV with survival outcomes in colorectal cancer patients. Relevant studies from PubMed, Embase and Web of Science were comprehensively examined up to August 20, 2022. Published language was not restricted during the search process. The MeSH term “pan-immune-inflammation value” was used to comprehensively identify potential studies. In addition, the references of the included studies were scanned for additional reports. The search was independently performed by two investigators (XC-Y and H-L).

### 2.2 Inclusion and exclusion criteria

The inclusion criteria were as follows:

(1) Studies examined the relationship between the PIV and long-term survival of patients with colorectal cancer;
(2) Hazard ratios (HRs) with 95% confidence intervals (CIs) were available;
(3) The cutoff value of the PIV was clearly reported.

The exclusion criteria were as follows:

(1) Studies were reported as case reports, reviews and letters;
(2) Duplicated data.

### 2.3 Data extraction and quality assessment

Two reviewers (XC-Y and H-L) conducted the data extraction independently and cross-checked all the results. The extracted data included first author, publication year, study interval, country, study design and sample size, selection method, cut-off value, clinicopathological features like age, sex and tumor stage, and survival data.

The quality of included studies were also evaluated following this method described by Lin et al. (19), which contains predefined nine items. A study could get a final score from 0 to 9 after assessment.

### 2.4 Outcomes

In the present study, the primary outcomes were to investigate the relationship between the PIV and long-term survival in patients with colorectal cancer. Long-term outcomes included OS and PFS. Of note, since disease-free survival (DFS), recurrence-free survival (RFS) and PFS share the similar endpoints, they were analyzed together as one outcome, PFS, as previously suggested (20).

### 2.5 Statistical analysis

The HRs with their 95% CIs were used as the effect size for OS and PFS. Statistical heterogeneity among enrolled studies was assessed using I^2^ statistic. All pooled analyses were conducted assuming the random-effects model, which accounts for variance across included studies. Subgroup analysis and sensitivity analysis were utilized to evaluate the credibility of pooled results. Begg’s funnel plot was applied to assess the possibility of publication bias. A two-tailed P value <0.05 was considered statistically significant. All of these statistical analyses were performed by Review Manager Software, version 5.3 (Cochrane, London, UK) and Stata, version 12.0 (Statacorp, College Station, TX).

## 3 Results

### 3.1 Study characteristics

**Figure 1:**
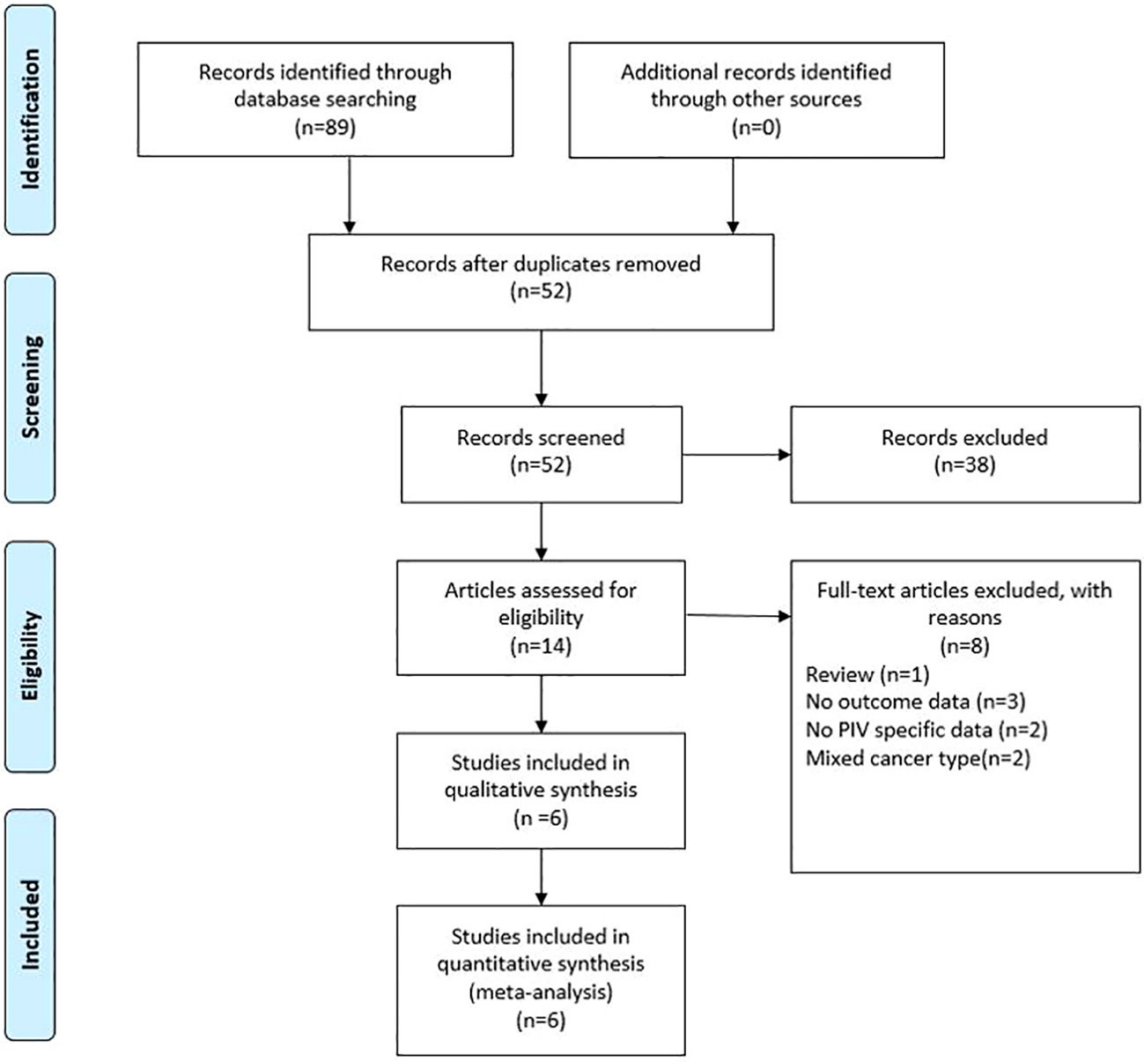
The PRISMA Flowchart of study selection.

**Figure 2:**
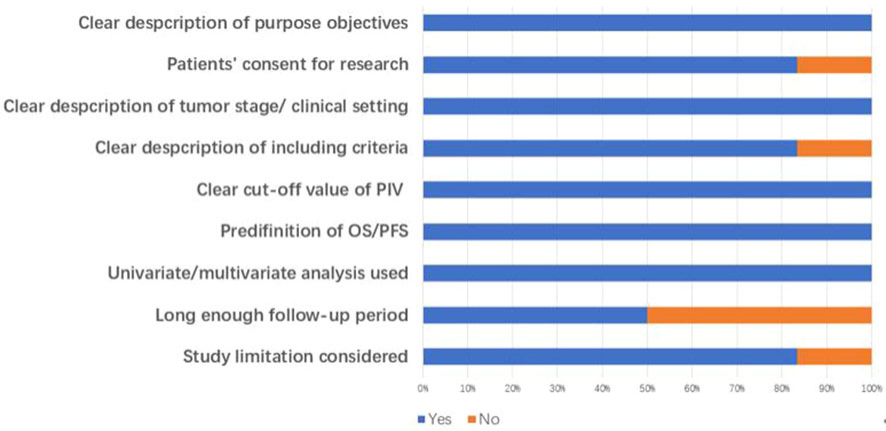
Quality assessment of included studies.

As shown in **Figure 1**, the search strategy yielded a total of 89 records. After careful title, abstract assessment and full text assessment, 6 studies (21–26) were finally included in the present study. The basic information of the included studies was shown in **Tables 1**, **2**. A total of 1879 patients from Italy (21, 23), Turkey (22), Spain (24) and Japan (25, 26) were included in this study. These studies were published from 2020 to 2022 with a sample size ranging from 86 to 758. Among these studies, two studies (21, 23) were designed as multicenter studies, and another four studies (22, 24–26) were single-center studies. In addition, five (21–24, 26) and six studies (21–26) reported the relationship between baseline PIV and OS and PFS, respectively; and two studies (21, 24) reported the relationship between early PIV increase after treatment initiation and OS and PFS, respectively. Moreover, the cut-off value of the PIV varies a lot among these studies, ranging from 209 to 492. The quality of the included studies was good with a median score of 8 (range: 6-9, **Figure 2** and **Table S1**).

**Table 1:** Basic information of included studies.

Reference	Country	Study design	Study interval	Sample size	Age, years	Sex(Male/Female)	Tumor stage	Survival analysis
Corti, 2021^[21]^	Italy	M; R	2014-2020	163	NR	90/73	Metastatic	OS; PFS
Efile, 2021 ^[22]^	Turkey	S; R	2008-2016	304	NR	NR	Non-metastatic	OS; DFS
Fuca, 2020 ^[23]^	Italy	M; R	2008-2018	438	62(IQR:53-68)	275/163	Metastatic	OS; PFS
Perez-Martelo, 2022 ^[24]^	Spain	S; R	2015-2018	130	68.8(range:26-88)	96/34	Metastatic	OS; PFS
Sato (1), 2022 ^[25]^	Japan	S; R	2013-2020	86	70(range:37-93)	50/36	Non-Metastatic	RFS
Sato (2), 2022 ^[26]^	Japan	S; R	2000-2019	758	NR	466/292	Non-metastatic	OS; RFS

R, retrospective; S, single center; M, multiple center; NOS, Newcastle Ottawa Scale; NR, not report; IQR, inter-quartile range.

OS, overall survival; PFS, progression-free survival; RFS, recurrence-free survival; DFS, disease-free survival.

**Table 2:** Survival information of included studies.

Reference	Sample size (High / Low)	Selection method	Cut-off value	Survival analysis	Median follow-up (months)	Analysis method	OSHR (95%CI)	RFS/PFS/DFSHR (95%CI)
Corti, 2021^[21]^	163(63:100)	MSR	492	OS; PFS	31	Multivariate	3.00 (1.49-6.04)	1.91 (1.06-3.44)
Efile, 2021 ^[22]^	304(152:152)	Median	491	OS; DFS	NR	Multivariate	2.43(1.55-3.79)	2.28 (1.51-3.45)
Fuca, 2020 ^[23]^	438(230:208)	MSR	380	OS; PFS	38.4(IQR:27.4-50.9)	Multivariate	1.55 (1.02–2.37)	1.53 (1.09–2.15)
Perez-Martelo, 2022 ^[24]^	130(70:60)	Literature	380	OS; PFS	NR	Multivariate	1.82 (1.15–2.90)	1.56 (1.05–2.31)
Sato (1), 2022 ^[25]^	86(63:23)	ROC	209	RFS	35(range:1-104)	Multivariate	NR	3.99 (1.69–9.45)
Sato (2), 2022 ^[26]^	758(190:568)	ROC	376	OS; RFS	63.5	Multivariate	2.49( 1.55–3.98)	1.70 (1.10–2.62)

OS, overall survival; PFS, progression-free survival; RFS, recurrence-free survival; DFS, disease-free survival; ROC, receiver operating characteristic curve. MSR, maximally selected rank; IQR, inter-quartile range NR, not report.

### 3.2 Relationship between baseline PIV and OS

**Figure 3:**
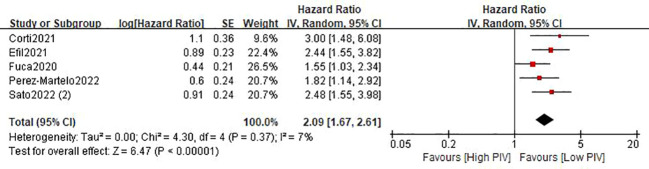
Forest plot assessing the relationship between PIV and OS.

Five studies (21–24, 26) involving 1793 patients described the association between the baseline PIV and OS. The pooled HR was 2.09 (95%CI: 1.67-2.61; P<0.0001; I^2 =^ 7%), which indicated that a high PIV was significantly associated with decreased OS in patients with colorectal cancer (**Figure 3** and **Table 3**). Furthermore, subgroup analyses based on country, study design, sample size, and tumor stage were performed. As shown in **Table 3** and **Figure S1**, the pooled results of all subgroup analyses revealed that patients in the high PIV group had a substantially reduced OS when compared with these in the low PIV group. Additionally, sensitivity analysis by deleting one study at a time showed that the pooled outcome did not substantially change (**Figure S3A**).

**Table 3:** Subgroup analyses for OS and PFS of PIV-high patients vs. PIV-low patients.

		Studies, n	Patients, n	HR (95%CI)	P value	I^2^ (%)
Overall survival						
	Total	5	1793	2.09(1.67-2.61)	<0.0001	7
Country	Eastern	1	758	2.48(1.55-3.98)	0.0001	–
	Western	4	1035	2.00(1.53-2.61)	<0.0001	17
Sample size	>200	3	1500	2.08(1.52-2.83)	<0.0001	32
	≤200	2	293	2.18(1.36-3.48)	0.001	25
Study design	Multi-center	2	601	2.02(1.07-3.82)	0.03	60
	Single-center	3	1192	2.23(1.71-2.92)	<0.0001	0
Tumor stage	Non-metastatic	2	1062	2.46(1.78-3.40)	<0.0001	0
	Metastatic	3	731	1.86(1.35-2.58)	0.0002	20
Progression-free survival						
	Total	6	1879	1.82(1.49-2.22)	<0.0001	15
Country	Eastern	2	844	2.39(1.06-5.40)	0.04	67
	Western	4	379	1.74(1.42-2.14)	<0.0001	0
Sample size	>200	3	1500	1.77(1.41-2.23)	<0.0001	6
	≤200	3	449	2.02(1.28-3.20)	0.003	46
Study design	Multi-center	2	601	1.62(1.21-2.17)	0.001	0
	Single-center	4	1278	1.97(1.46-2.64)	<0.0001	36
Tumor stage	Non-metastatic	3	1148	2.20(1.51-3.20)	<0.0001	37
	Metastatic	3	731	1.60(1.27-2.02)	<0.0001	0

### 3.3 Relationship between baseline PIV and PFS

**Figure 4:**
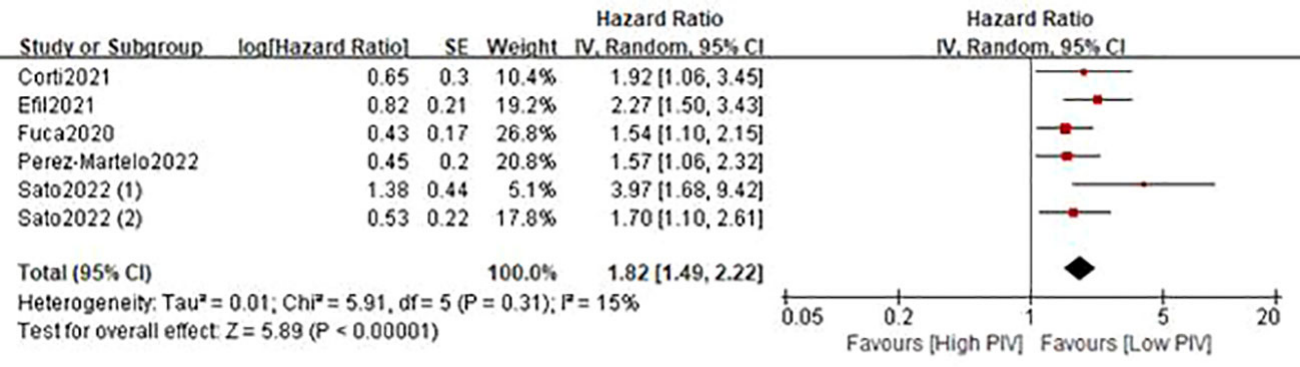
Forest plot accessing the relationship between PIV and PFS.

A total of six studies (21–26) involving 1879 patients reported on PFS. The pooled HR was 1.82 (95%CI: 1.49-2.22; P<0.0001; I^2 =^ 15%), which suggested that patients in the high PIV group had a worse PFS when compared with patients in the low PIV group (**Figure 4** and **Table 3**). Similarly, subgroup analyses based on country, study design, sample size, and tumor stage demonstrated that the pooled results remained consistent in each subgroup (**Table 3** and **Figure S2**). Sensitivity analysis showed that the combined effect was not significantly changed (**Figure S3B**).

### 3.4 Relationship between early PIV increase and OS/PFS

**Figure 5:**
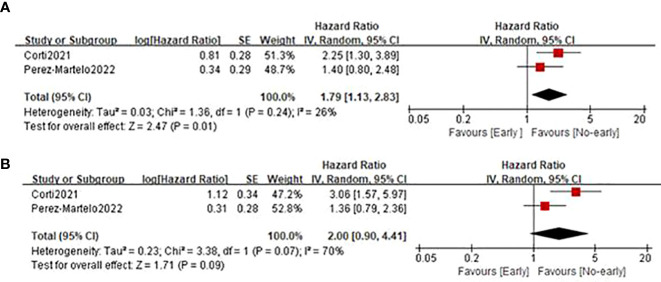
Forest plot assessing the relationship between PIV dynamics and survival outcomes including OS **(A)** and PFS **(B)**.

Only two studies (21, 24) involving 277 cases reported the relationship between early PIV increase after the treatment initiation and survival outcomes. As shown in **Figure 5**, the combined results suggested that early PIV increase was substantially correlated with decreased OS (HR=1.79; 95%CI: 1.13-2.93; P=0.01; I^2 =^ 26%), and a trend toward poor PFS (HR=2.00; 95%CI: 0.90-4.41; P=0.09; I^2 =^ 70%).

### 3.5 Publication bias

The Begg’s funnel plot was performed to assess the possibility of publication bias. As shown in **Figure S4**, the funnel plots of OS and PFS were symmetric, and the P values of Begg’s test were 0.130 and 0.060, respectively, indicating that these pooled outcomes were absence of publication bias.

## 4 Discussion

In 2020, Fuca et al. (23) first developed the PIV based on commonly used peripheral blood count parameters as a systemic inflammation-related prognostic biomarker for metastatic colorectal cancer. Since then, the PIV has been widely used as a cheap, readily available and reliable index to evaluate the prognosis of various cancers (27–29). In the present study, we included six studies with 1879 patients with colorectal cancer and found that high PIV was significantly associated with decreased OS and PFS. Meanwhile, we have further identified that the early PIV increase after the treatment initiation was also associated with significantly poor OS and a trend toward worse PFS in colorectal cancer patients. Therefore, the PIV may have a good discriminatory value and remains an effective inflammatory index for predicting long-term survival outcomes in colorectal cancer.

Systemic inflammatory reflection has been well confirmed to be closely associated with the occurrence and progression of malignancies (5). Increased neutrophils and monocytes in the tumor microenvironment have been reported to induce myeloid-derived suppressor cells, thereby suppressing the host immunity and prompting the tumor growth (30, 31). In addition, monocytes can differentiate into tumor-associated macrophages, which is associated with creating a favorable microenvironment for cancer development (32). Platelets are reported to secrete TGF-β, FGF and VEGF, which contribute to the epithelial–mesenchymal transition and angiogenic process (33, 34). Moreover, the interaction between platelets and tumor cells recruits and activates neutrophils and monocytes, which is required for the formation of distal metastasis sites (34). While lymphocytes, especially cytotoxic T lymphocytes, as the most important cell-mediated anti-tumor immune cells, inhibit tumor cell proliferation and metastasis by inducing the lysis and apoptosis of tumor cells (35, 36). Low lymphocyte counts have been demonstrated to lead to poor prognosis in colorectal cancer patients (37). Reasonably, the PIV, combined with neutrophils, monocytes, lymphocytes, and platelets, may enable better understanding of the functional state of patients and predict the prognosis of patients with colorectal cancer.

In our combined analysis involving 1793 samples, we identified that the baseline PIV is an independent prognostic factor of OS in patients with colorectal cancer. Furthermore, subgroup analyses based on country, study design, sample size and tumor stage showed our results were consistent and robust. Meanwhile, the sensitivity analysis showed that there was no significant change in the correlation between high PIV and decreased OS. Additionally, we have further investigated the relationship between the PIV and PFS. The pooled result including 1879 patients showed that patients in the high PIV group has a substantially decreased PFS. Similarly, the subgroup analyses and sensitivity analysis supported the reliability of this incorporated result. Furthermore, we have also preliminarily explored the relationship between the early PIV increase after the treatment initiation and survival outcomes. The integrated results showed that the early PIV increase was correlated with decreased OS and tended to have a poor PFS. However, given that there were only two studies with small samples included, these results should be interpreted with caution and more studies with big sample size were required to further classify this issue. Based on these results, the PIV may be regarded as an effective prognostic indicator of long-term results of colorectal cancer.

There are some limitations to be noted in the present study. First, all involved studies were retrospective in nature, which may increase the risk of bias, and more prospective studies and randomized controlled trials are required to further investigate this issue. Second, due to the limited number of included studies, the value of the PIV dynamics in survival outcomes needs to be further clarified. Third, the cut-off value of PIV varies greatly among studies, which might affect the clinical utility of these findings. ​Finally, we were also unable to compare the prognostic predictability of PIV with other biomarkers in colorectal cancer patients, with few data eligible.

## 5 Conclusions

The findings of the meta-analysis suggested that the PIV is of great significance in predicting long-term survival results in patients with colorectal cancer. However, further research is still required to validate the value of PIV in colorectal malignancy.

## Author contributions

X-CY wrote the manuscript. X-CY and HL performed the data search and data analysis. X-CY and HL and D-CL prepared figures and tables. All authors reviewed the manuscript. X-WL and R-HC approved the final manuscript.
