# Supplementary material for: Prognostic value of pan-immune-inflammation value in colorectal cancer patients: A systematic review and meta-analysis
Source: Front Oncol. 2022 Dec 22;12:1036890. doi: 10.3389/fonc.2022.1036890 (PMC9813847; doi:10.3389/fonc.2022.1036890)
Supplement: Supplementary file 1 [file DataSheet_1.docx]

Table S1. Quality assessment of included studies.

| **Items** | **Corti,2021 ^[21]^** | **Efile,2021 ^[22]^** | **Fuca,2020 ^[23]^** | **Perez‑Martelo,2022 ^[24]^** | **Sato,2022 (1) ^[25]^** | **Sato,2022 (2) ^[26]^** |
| --- | --- | --- | --- | --- | --- | --- |
| Study limitation considered | 1 | 0 | 1 | 1 | 1 | 1 |
| Long enough follow-up period | 0 | 1 | 1 | 1 | 0 | 0 |
| Univariate/multivariate analysis used | 1 | 1 | 1 | 1 | 1 | 1 |
| Predefinition of OS/PFS | 1 | 1 | 1 | 1 | 1 | 1 |
| Clear cut-off value of PIV | 1 | 1 | 1 | 1 | 1 | 1 |
| Clear description of including criteria | 1 | 0 | 1 | 1 | 1 | 1 |
| Clear description of tumor stage/ clinical setting | 1 | 1 | 1 | 1 | 1 | 1 |
| Patients' consent for research | 1 | 0 | 1 | 1 | 1 | 1 |
| Clear description of purpose objectives | 1 | 1 | 1 | 1 | 1 | 1 |


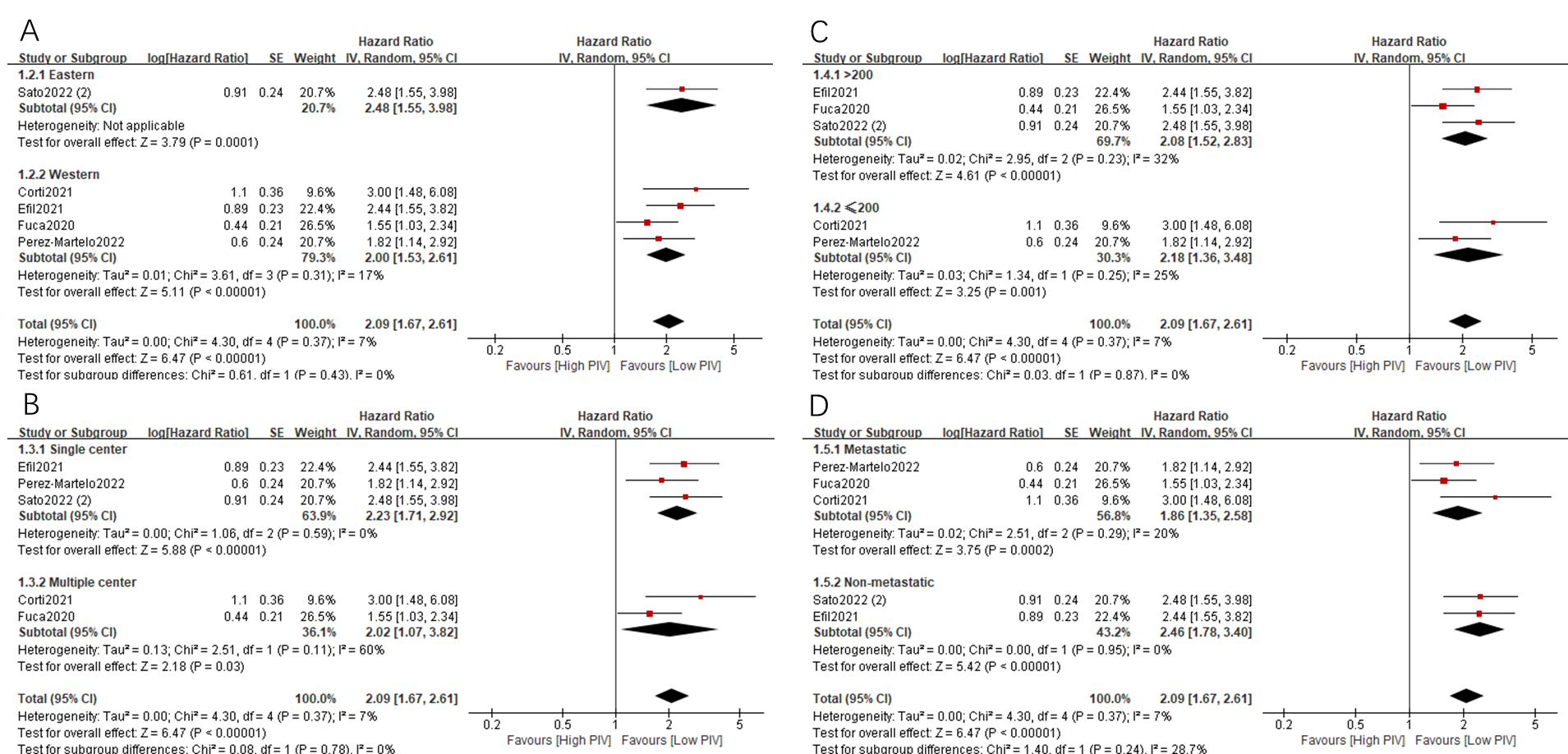


Figure S1. Forest plot of subgroup analyses assessing the relationship between PIV and OS. A: Country (eastern vs. western); B: Study design (multiple-center vs. single-center) C: Sample size (>200 vs. ≤200); D: Tumor stage (non-metastatic vs. metastatic).


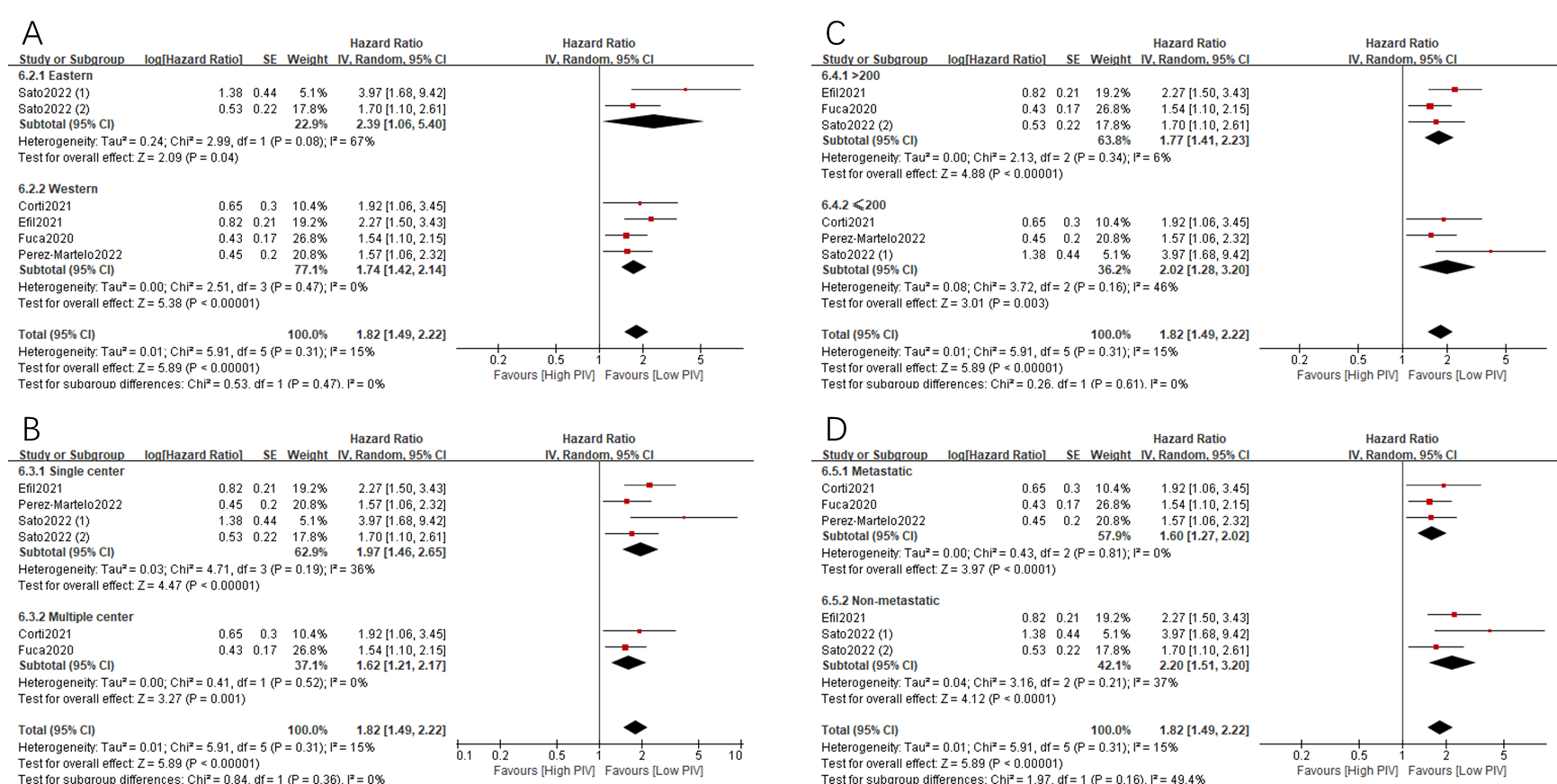


Figure S2. Forest plot of subgroup analyses assessing the relationship between PIV and PFS. A: Country (eastern vs. western); B: Study design (multiple-center vs. single-center) C: Sample size (>200 vs. ≤200); D: Tumor stage (non-metastatic vs. metastatic).


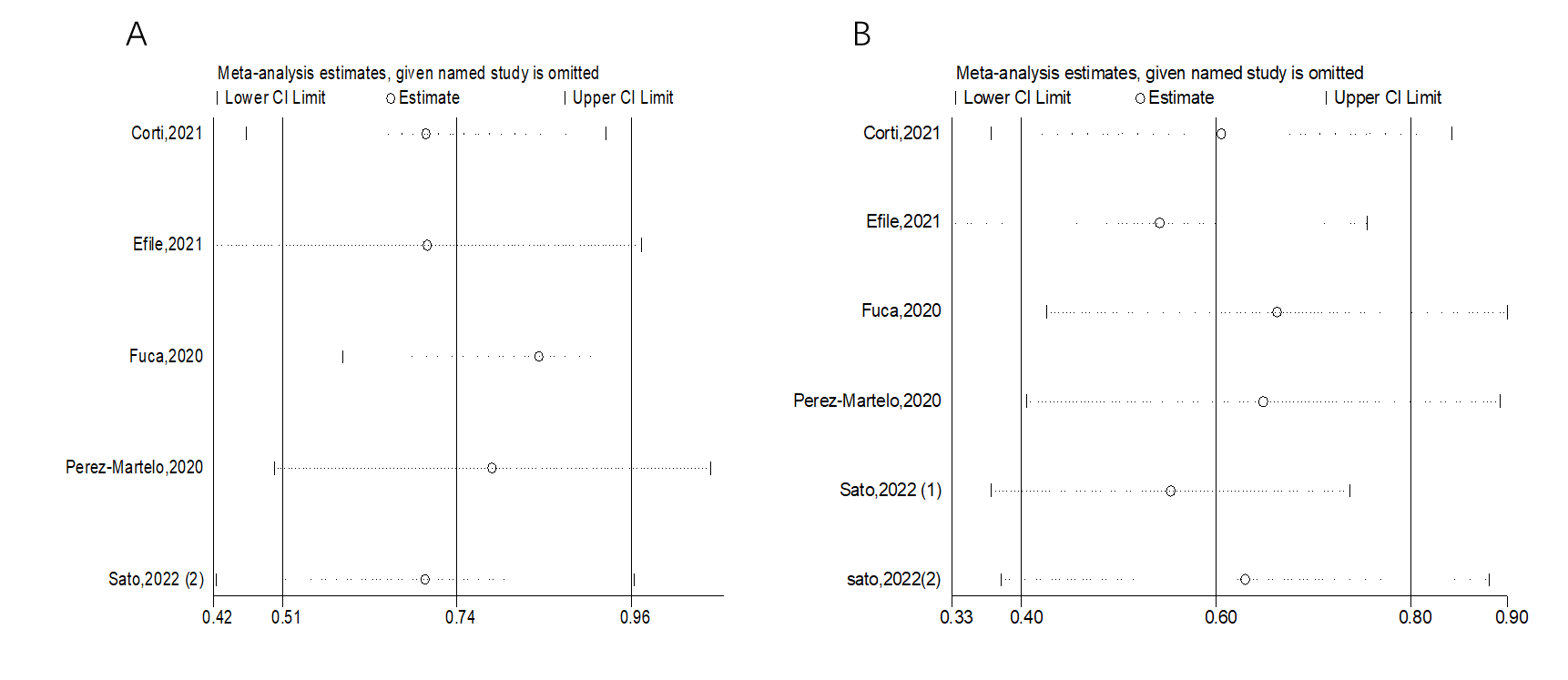


Figure S3. Sensitivity analyses assessing the relationship between PIV and survival outcomes including OS (A) and PFS (B).


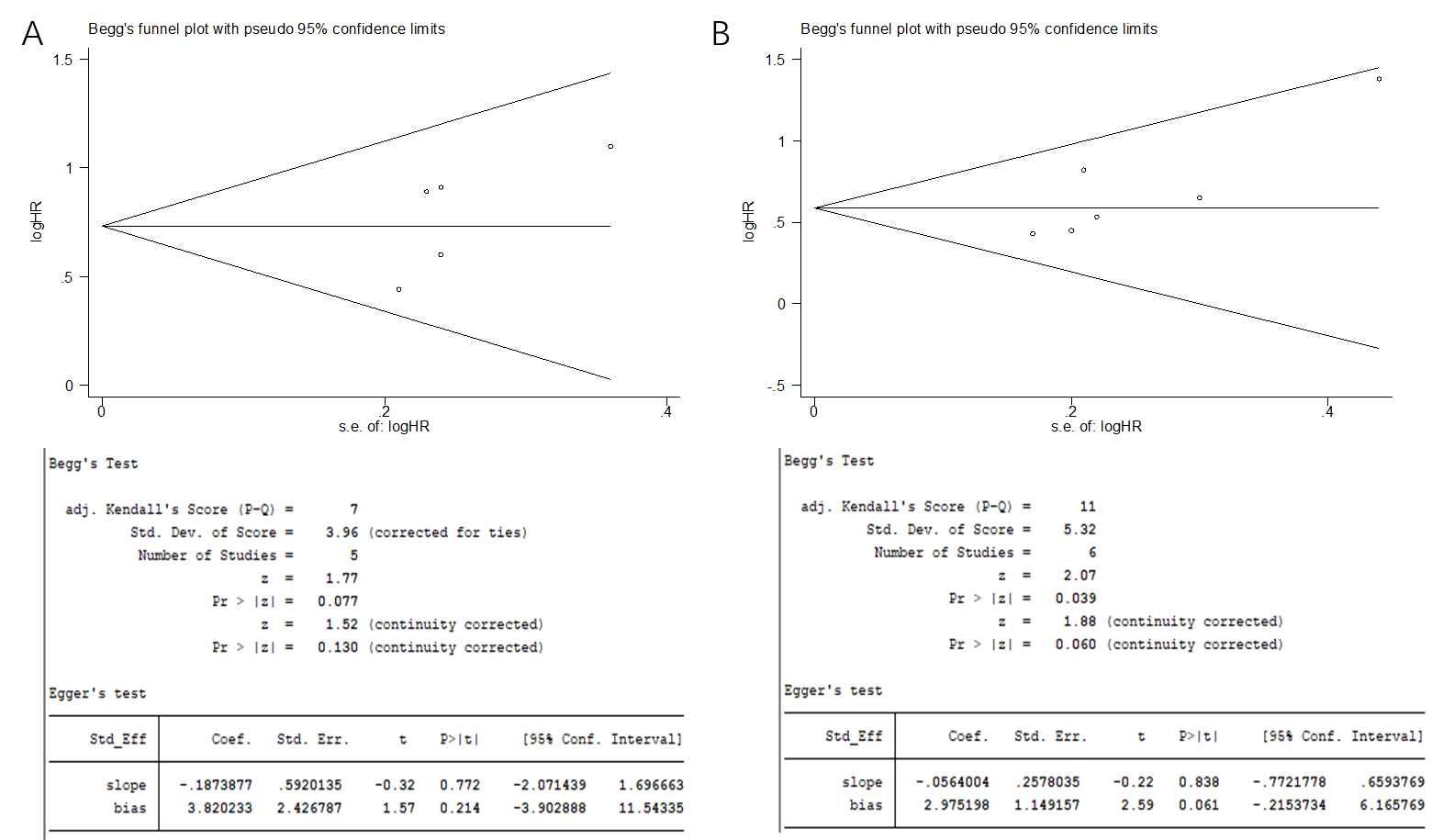


Figure S4. Begg’s funnel plot assessing publication bias between PIV and survival outcomes, including OS (A) and PFS (B). The Begg’s P values were 0.130 and 0.060, respectively.
